# B-Cell Lymphoma 6 (BCL6) Is a Host Restriction Factor That Can Suppress HBV Gene Expression and Modulate Immune Responses

**DOI:** 10.3389/fmicb.2018.03253

**Published:** 2019-01-10

**Authors:** Chun-Ta Lin, Yue-Ting Hsieh, Yeng-Jey Yang, Shih-Hui Chen, Cheng-Hsuan Wu, Lih-Hwa Hwang

**Affiliations:** ^1^Institute of Microbiology and Immunology, National Yang-Ming University, Taipei, Taiwan; ^2^Biomedical Industry Ph.D. Program, National Yang-Ming University, Taipei, Taiwan

**Keywords:** Hepatitis B virus (HBV), B-cell lymphoma 6 (BCL6), restriction factor, transcriptional repressor, HBV clearance

## Abstract

Hepatitis B virus (HBV) infection causes acute and chronic liver inflammation. Recent studies have demonstrated that some viral antigens can suppress host innate and adaptive immunity, and thus lead to HBV liver persistency. However, the cellular factors that can help host cells to clear HBV during acute infection remain largely unknown. Here, we used HBV-cleared and HBV-persistent mouse models to seek for cellular factors that might participate in HBV clearance. HBV replicon DNA was delivered into the mouse liver by hydrodynamic injection. RNA-Seq analysis was conducted to identify immune-related genes that were differentially expressed in HBV-persistent and HBV-cleared mouse models. A cellular factor, B cell lymphoma 6 (BCL6), was found to be significantly upregulated in the liver of HBV-cleared mice upon HBV clearance. Co-expression of BCL6 and a persistent HBV clone rendered the clone largely cleared, implicating an important role of BCL6 in controlling HBV clearance. Mechanistic studies demonstrated that BCL6 functioned as a repressor, binding to and suppressing the activities of the four HBV promoters. Correspondingly, BCL6 expression significantly reduced the levels of HBV viral RNA, DNA, and proteins. BCL6 expression could be stimulated by inflammatory cytokines such as TNF-α; the BCL6 in turn synergized TNF-α signaling to produce large amounts of CXCL9 and CXCL10, leading to increased infiltrating immune cells and elevated cytokine levels in the liver. Thus, positive feedback loops on BCL6 expression and immune responses could be produced. Together, our results demonstrate that BCL6 is a novel host restriction factor that exerts both anti-HBV and immunomodulatory activities. Induction of BCL6 in the liver may ultimately assist host immune responses to clear HBV.

## Introduction

Hepatitis B virus (HBV) infection may cause severe diseases in the liver such as hepatitis, liver cirrhosis, and hepatocellular carcinoma (Xu et al., 2014). According to an estimation by the World Health Organization (WHO), ~257 million people are chronically infected with HBV and ~1 million patients die from HBV-associated diseases each year (Lozano et al., 2012; Ott et al., 2012). Although public health policy and a preventive vaccine have provided effective blockage of HBV transmission, there is no effective therapy to cure chronic HBV infection. Currently, two types of drugs are being used for the treatment of HBV infection in the clinic, interferon (IFN), and nucleoside/nucleotide analogs (Tang et al., 2014; Gill and Kennedy, 2015). However, only a few patients can reach the HBV surface antigen (HBsAg) seroclearance stage after receiving therapy, and they are still at risk of viral rebound once patients' immunities are suppressed. The major difficulties for curing chronic HBV infection are the exhaustion and depletion of HBV-specific immune responses (see review in Ferrari, 2015) and the persistence of covalently closed circular DNA (cccDNA) in hepatocyte nuclei (Moraleda et al., 1997; Dandri et al., 2000).

More than 90% of people acutely infected with HBV will recover from the infection, but 5–10% of adults and most neonates fail to clear the virus and progress to chronic infection. Recently, growing evidence has indicated that HBsAg and HBV e antigen (HBeAg) can tolerize host immunity and assist HBV in establishing liver persistency (see review in Tsai et al., 2018). These two viral antigens are secretory proteins and can suppress both innate and adaptive immune responses. For example, it has been shown that HBsAg suppresses Toll-like receptor (TLR) 3 and TLR4-induced type I interferon (IFN) production in liver non-parenchymal cells, the major intrahepatic innate immune cells, and thus downregulates the activation of adaptive immunity (Wu et al., 2009). Conversely, blocking circulating HBsAg with an antibody in an HBV-persistent mouse model significantly restores the immune responses against HBV after receiving a therapeutic vaccine (Zhu et al., 2016). Additionally, maternally-derived HBeAg in neonates can skew the function of macrophages toward M2 phenotypes and help to establish HBV persistency in offspring (Tian et al., 2016). While the viral factors governing HBV persistence are gradually being understood, the cellular factors regulating host immune responses or involved in viral clearance during acute infection remain largely unknown.

We have previously established an HBV-persistent mouse model in immunocompetent FVB/N mice through hydrodynamic injection (HDI) using HBV DNAs from clinical isolates (Chen et al., 2012). Interestingly, injection of two relevant HBV clones, B6.2 and B6.2S, which differ only by one amino acid in the HBsAg region, resulted in opposing effects; whereas B6.2 remained persistent, B6.2S was gradually cleared from the FVB/N mouse liver within 18 weeks. To investigate the mechanisms accounting for this difference, in this study we have conducted RNA-Seq analysis on the liver RNAs from these two groups of mice. We have identified a cellular factor, B cell lymphoma 6 (BCL6), whose levels were significantly upregulated in the B6.2S-injected mouse liver during HBV clearance. BCL6 is a transcriptional repressor belonging to the broad complex, tramtrack, bric á brac-Poxvirus Zinc finger (BTB-POZ) protein family that represses gene transcription by recruiting diverse co-repressor complexes (Wong and Privalsky, 1998; Lemercier et al., 2002). BCL6 plays a pivotal role during mature B cell differentiation in germinal centers (GCs) (Dent et al., 1997; Fukuda et al., 1997) and is also involved in the development of follicular helper T (T_FH_) cells (Nurieva et al., 2009; Yu et al., 2009). BCL6 can dampen inflammatory activities in various cell types (Takeda et al., 2003; Igoillo-Esteve et al., 2011). However, a recent report showed that BCL6 functioned as a negative regulator of anti-viral responses in vesicular stomatitis virus (VSV)-infected RAW264.7 cells by preventing the transcription of IFN regulatory factor 7 (*Irf7*), which unexpectedly resulted in enhanced proinflammatory activity (Xu et al., 2016). Thus, BCL6 may exert anti-inflammatory or proinflammatory activities depending on cell types and/or stimuli. Despite these findings, the roles of BCL6 in HBV clearance have never been reported before.

In this study, we demonstrated that BCL6 functioned as a repressor of HBV promoters, which was independent of cell type or HBV genotype. BCL6 also acted as an immune modulator in hepatocytes to enhance chemokine production, leading to increased immune cell infiltration into the liver. For the first time, our studies identified BCL6 as a novel host restriction factor against HBV replication.

## Materials and Methods

### Animal Experiments

FVB/N mice were purchased from the National Laboratory Animal Center (Taipei, Taiwan) and housed in a bio-safety level 2 (BSL-2) animal room at the Animal Center of National Yang-Ming University (NYMU). Hydrodynamic injection was performed under anesthesia conditions for the animals and DNA solution equivalent to 8% of the mouse body weight was injected through the tail vein within 6–8 s. Serum levels of HBsAg and HBeAg were determined at the indicated time points post-DNA injection using HBsAg II and HBeAg kits (Roche, Switzerland) and measured by an automated analyzer (Roche Cobas 6000).

### Ethics Statement

The animal experiments were conducted with the approval of the Institutional Animal Care and Use Committee of National Yang-Ming University (Approval No. 1021230). All animal procedures involving the care and use of mice were performed following the “Guidelines for Care and Use of Laboratory Animals” and the “Animal Protection Act,” Council of Agriculture, Taiwan.

### RNA-Seq Analysis

Six B6.2 DNA-injected mice and 4 B6.2S DNA-injected mice were sacrificed at the time when B6.2S DNA was being cleared, which was defined by the serum levels of HBeAg dropping down to 1–20 COI. Total RNA was extracted from mouse livers by using the TruSeq mRNA library Prep (Illumina, USA). For each individual group, liver RNAs from each mouse were mixed at an equal ratio and then subjected to sequencing using an Illumina HiSeq 2000. The RNA-Seq reads were first mapped to the mouse reference genome (MGSCv37/ NCBI mm9) using TopHat2 and the expression of immune-related genes with ≥4 (or ≤0.25)-fold change between B6.2-injected mice and B6.2S-injected mice were selected for further analysis.

### Plasmid Construction

The construction of the pHBV1.3-B6.2 (B6.2 in short) and pHBV1.3-B6.2S (B6.2S in short) replicon plasmids, which included the 1.3-fold over-length HBV DNA (from a genotype B clinical isolate) in the pGEM4Z vector, has been described previously (Chen et al., 2012). To the generate *Bcl6*-expressing plasmids, the cDNAs encoding human or mouse BCL6 were PCR amplified from Huh-7 cells and the FVB/N mouse liver, respectively, and then cloned into pcDNA3.1 (Thermo Fisher Scientific, USA) and pORF (Invivogen, USA), respectively. The HBV replicons of different genotypes and the luciferase reporters under the control of different HBV promoters were kindly provided by Dr. Hui-Lin Wu (Hepatitis Research Center, National Taiwan University Hospital, Taiwan), including the core promoter (HBV: 1584-1888), pre-S1 promoter (2505-2847), pre-S2 promoter (2828-3223/1-157), and X promoter (1041-1433). The sequences of all the constructs were confirmed by DNA sequencing.

### Cell Cultures

Huh-7 (obtained from Dr. Pei-Jer Chen, National Taiwan University College of Medicine) and HepG2 (ATCC HB-8065) cell lines were cultured in Dulbecco's modified Eagle's medium (DMEM) supplemented with 10% fetal bovine serum, 2 mM L-glutamine, penicillin (100 U/ml), streptomycin (0.1 mg/ml) and amphotericin (0.25 μg/ml). AML12 cells (ATCC CRL-2254), an immortalized mouse hepatocyte cell line, were cultured in the same medium with additional 1% non-essential amino acid, 1% insulin-transferrin-selenium-ethanolamine and 0.1 μM dexamethasone.

### Reagents

Serum levels of HBsAg and HBeAg in the DNA-injected mice were determined by HBsAg II kit (Cat:04687787190, Roche, Switzerland) and HBeAg kit (11820583122, Roche), respectively and measured by an automated analyzer (Roche Cobas 6000). DNAs transfection in the cells was performed by Lipofectamine^TM^ 3000 (Invitrogen) according to the manufacturer's instructions. For western blot analysis, cells were lysed with RIPA buffer (50 mM Tris-HCl, pH 7.4, 150 mM NaCl, 1% NP40, 0.1% SDS, 0.1% deoxycholate and 2 mM EDTA) containing protease inhibitors (5 μg/ml aprotinin, 10 μg/ml leupeptin, 1 mM PMSF) and the antibodies used included anti-HBcAg (LTK BioLaboratories, Taiwan), anti-HBsAg (A10F1, kindly provided by Dr. Sheng-Chung Lee, National Taiwan University, Taiwan), anti-BCL6 (sc-858, Santa Cruz, USA), anti-β-actin (A5441, Sigma, USA) and horse radish peroxidase (HRP)-conjugated sheep anti-rabbit, or anti-mouse IgG (GE healthcare, USA). The cytokines used for stimulating *Bcl6* expression or chemokine induction included TNF-α (315-01A, PeProtech, USA) and IFN-γ (485MI, R&D, USA). The luciferase activity was measured by Dual-Glo® Luciferase Assay System (Promega, USA) following the manufacturer's instructions.

### Immunohistochemistry

Liver tissues were collected from the mice at the indicated time points. IHC staining was performed on 5-μm paraffin sections with rabbit anti-HBcAg (B0586, DAKO, Denmark) and anti-BCL6 (sc-858, Santa Cruz) antibodies and then developed using the Envision System-HRP, DAB (DAKO) following the manufacturer's instructions. The liver sections were counterstained with hematoxylin.

### RNA Extraction and Reverse Transcription-Quantitative PCR (RT-qPCR) Analysis

RNA from homogenized livers or cell lines was extracted using TRIzol^TM^ (Invitrogen), and 2 μg of the RNA were treated with DNase I (M6101, Promega) for 30 min and then subjected to reverse transcription. One-twentieth of the cDNA products was analyzed using a StepOnePlus^TM^ Real-Time PCR system (Thermo Fisher Scientific). Fast SYBR® Green Master Mix (4385616, Invitrogen) was used according to the manufacturer's instructions. The sequences of the primers used for the PCR are shown in Supplementary Table S1.

### Extraction of Intracellular Core-Associated HBV DNA

The cells transfected with HBV replicon DNA were lysed in NET buffer (100 mM NaCl, 50 mM Tris-HCl, pH 8.0, 0.5% NP-40 and 1 mM EDTA). After removal of the nuclear pellet by centrifugation at 13,000 x g at 4°C for 20 min, the plasmid DNA in the supernatant was first digested with micrococcal nuclease (Thermo Fisher Scientific) at 37°C for 30 min. The HBV core complexes were subsequently disrupted by protease K in a buffer containing 0.5% SDS at 55°C overnight. Viral DNA was subjected to phenol/chloroform extraction and precipitated with isopropanol.

### Southern Blot and Northern Blot Analyses

Southern blot and Northern blot analyses were performed mainly following the protocol described in “DIG Application Manual for Filter Hybridization” from Roche. Briefly, the RNA or intracellular core-associated HBV DNA extracted from Huh-7 cells were separated on a 1% agarose gel in 0.01 M Na^+^-phosphate buffer (pH 7.0 for Northern blot) or in 0.5X TAE buffer (20 mM Tris-base, 10 mM acetic acid, and 0.5 mM EDTA for Southern blot). The gel was blotted onto a positively charged nylon membrane (Cat: 11417240001, Roche), which was then hybridized with a digoxigenin (DIG)-labeled probe encompassing the HBx coding region (nt 1372–1833) that was generated using the DIG PCR Probe Synthesis kit (Cat: 1636090910, Roche). After 16 h of hybridization at 45°C (Southern blot) or 52°C (Northern blot), the membrane was washed following the instructions and blocked with 1X DIG blocking buffer (Cat: 11096176001, Roche) for 30 min and then incubated with alkaline phosphatase (AP)-conjugated anti-DIG antibody (1:10,000 dilution, Cat: 11093274910, Roche). The signals were detected by chemiluminescence using the CDP-*Star* substrate (Cat: 2041677, Roche).

### Chromatin Immunoprecipitation (ChIP)

ChIP was performed following a protocol from Abcam. Huh-7 cells were transfected with the indicated DNA using the Lipofectamine^TM^ 3000 reagent. Forty-eight hours post-transfection, ~5 x 10^6^ cells were used for one immunoprecipitation experiment. The cells were fixed with 1% formaldehyde at room temperature for 10 min and quenched with 125 mM glycine for another 5 min. After washing with PBS, the cells were lysed in ChIP lysis buffer (50 mM HEPES, pH 7.5, 140 mM NaCl, 1 mM EDTA, 1% Triton X-100, 0.1% sodium deoxycholate, 1% SDS, 5 μg/ml aprotinin, 10 μg/ml leupeptin, and 1 mM PMSF) and incubated on ice for 10 min. The cells were subjected to sonication to shear DNA fragments into small sizes ranging from 200 to 500 bp. The sonicated cell lysates were diluted to 0.2% SDS and were incubated with anti-FLAG antibody (M2, Sigma) or IgG control antibody overnight at 4°C. Protein G Mag Sepharose beads (28967066, GE Healthcare) were used to pull down the antibody-protein-DNA complexes, which were then washed as follows: twice with low salt wash buffer (20 mM Tris-HCl, pH 8.0, 0.1% SDS, 1% Triton X-100, 2 mM EDTA, and 150 mM NaCl), twice with high salt wash buffer (20 mM Tris-HCl, pH 8.0, 0.1% SDS, 1% Triton X-100, 2 mM EDTA, and 500 mM NaCl) and twice with LiCl wash buffer (10 mM Tris-HCl, pH 8.0, 1% NP-40, 0.25 m LiCl, 1% sodium deoxycholate, and 1 mM EDTA). The immunoprecipitates were eluted with elution buffer (1% SDS and 100 mM NaHCO_3_) and the cross-links were reverted by heating the samples at 65°C for overnight. The samples were then treated with protease K and the DNA was purified by phenol/chloroform extraction for qPCR analysis.

### Statistical Analysis

All of the statistical analyses were performed by GraphPad Prism version 7.0c for Mac OS X (GraphPad Software, La Jolla California USA). The method used for each experiment is described in the figure legends. The asterisks indicate statistical significance (^*^P < 0.05, ^**^P < 0.01, and ^***^P < 0.001).

## Results

### Intrahepatic Levels of BCL6 Are Upregulated in HBV-Cleared Mice During the HBV Clearance Stage

**Figure 1:**
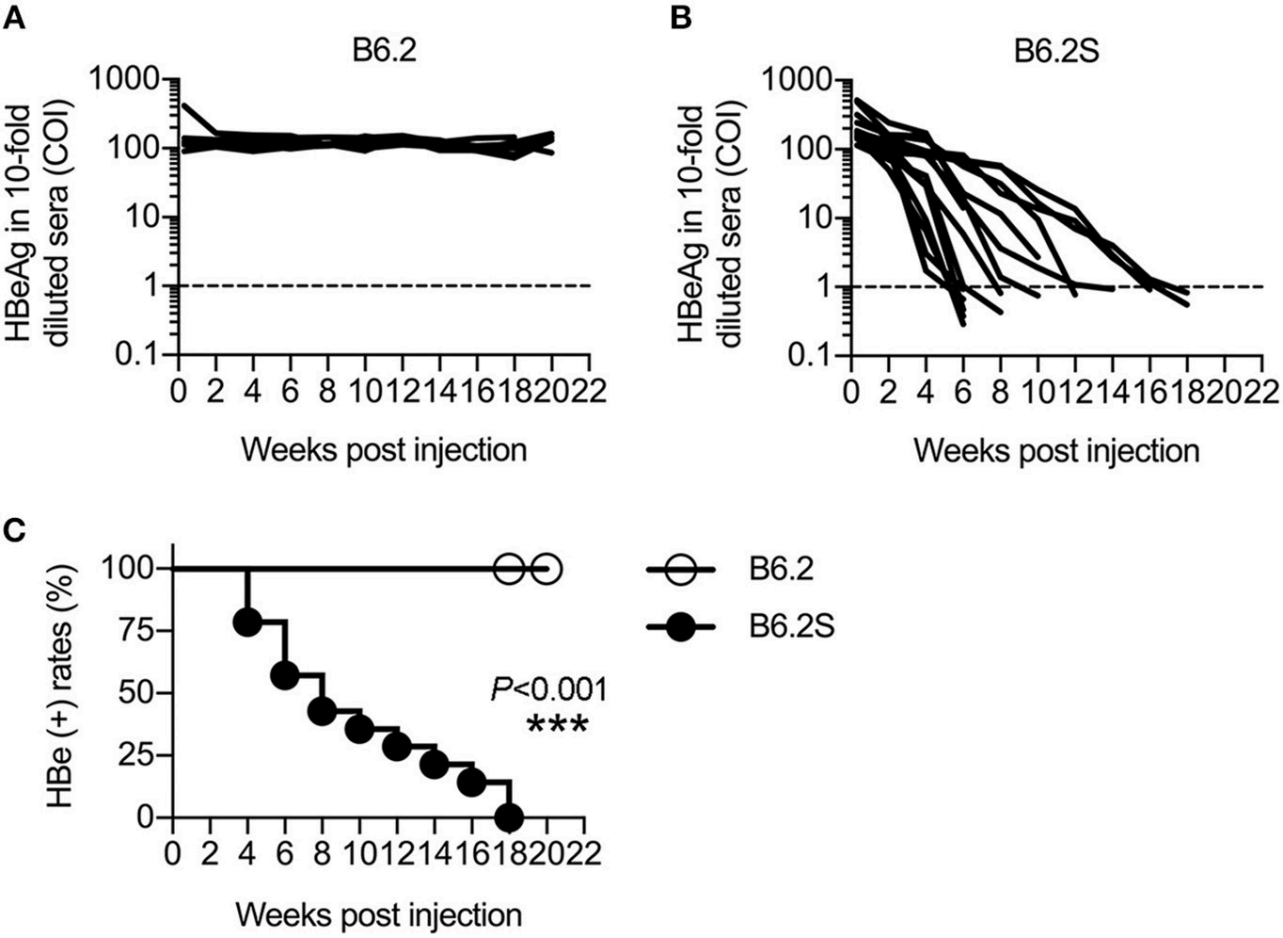
HBV replicons B6.2 and B6.2S exhibit different persistence rates in FVB/N mice. Ten micrograms of **(A)** B6.2 or **(B)** B6.2S HBV replicon DNA were delivered into the liver of FVB/N mice through HDI. The serum levels of HBeAg were measured periodically to monitor HBV persistence in the liver. Each line represents one mouse. The levels of HBeAg in 10-fold diluted sera were expressed as the cut-off index (COI) and the dotted lines indicate the cut-off value for HBeAg. **(C)** The kinetics of HBeAg clearance were monitored over a period of 22 weeks post-DNA injection. The HBeAg (+) rates for the B6.2 and B6.2S groups were compared using the log-rank test (***P < 0.001, *N* = 7 in the B6.2 group and *N* = 12 in the B6.2S group).

**Figure 2:**
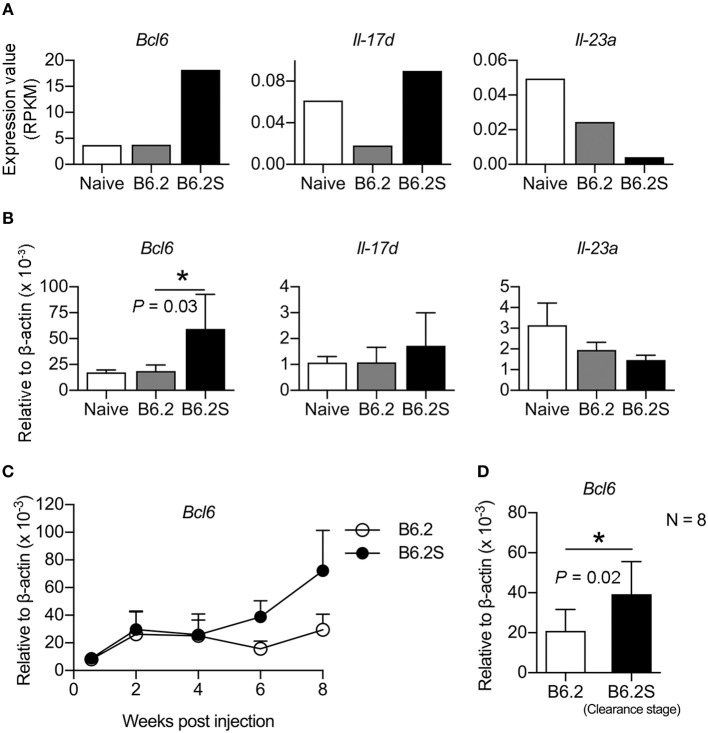
BCL6 levels are upregulated in the mouse liver during the HBV clearance stage. Liver RNAs extracted from B6.2- or B6.2S-injected mice were examined by **(A)** RNA-Seq analysis or **(B)** RT-qPCR analysis. RPKM means the reads per kilobases per million reads (*N* = 6 for B6.2 and *N* = 4 for B6.2S). **(C)** The expression profiles of *Bcl6* in the liver of HBV DNA-injected mice are shown over a period of 8 weeks post-DNA injection. *Bcl6* expression was determined by RT-qPCR (*N* = 3 for each time point). **(D)** RT-qPCR analysis of *Bcl6* expression was conducted on another set of RNAs prepared from the B6.2S-injected mouse livers (*N* = 8) during the HBV clearance stage and from the B6.2-injected mice at equivalent time points. The data presented are the mean ± standard deviation (SD). *P < 0.05 for one-way ANOVA and the two-tailed Student's *t*-test in **(B,D)**, respectively.

We previously constructed two HBV replicons, pHBV1.3-B6.2 (B6.2 in short) and pHBV1.3-B6.2S (B6.2S) which only had an N214S mutation in the HBsAg region compared to B6.2, and introduced them into the liver of FVB/N mice by HDI to compare their persistence rates in the mice (Chen et al., 2012). Serum levels of HBeAg were measured as a surrogate marker to monitor the presence of HBV DNA in the liver. Interestingly, we found that the B6.2 clone remained persistent in the mouse liver for up to 22 weeks (Figure 1A), whereas the B6.2S clone was gradually cleared from the mouse liver from 6 to 18 weeks post-DNA injection (w.p.i.) (Figure 1B). The difference in HBeAg (+) rates between these two groups of mice was statistically significant (*P* < 0.001, Figure 1C). Using these two mouse models, we sought to identify host factors that might be involved in HBV clearance. We performed RNA-Seq analysis on the liver RNAs from these two groups of mice (*N* = 4–6) isolated at the time when HBV was being cleared in B6.2S animals, defined by the cut-off index (COI) values of HBeAg in 10-fold diluted sera reaching 1–20 (Figure 1B), which was ~6–12 w.p.i. Many genes were found to be differentially expressed in these two groups of mice; however, we only focused on immune-related genes that had more than 4-fold differences in their expression levels. The following three genes were identified: B-cell lymphoma 6 (*Bcl6*), interleukin 17D (*Il-17d*), and *Il-23a* (Figure 2A). However, the RT-qPCR validation results demonstrated that only *Bcl6* showed a significantly different expression pattern (Figure 2B). Thus, *Bcl6* was used for further experiments.

**Figure 3:**
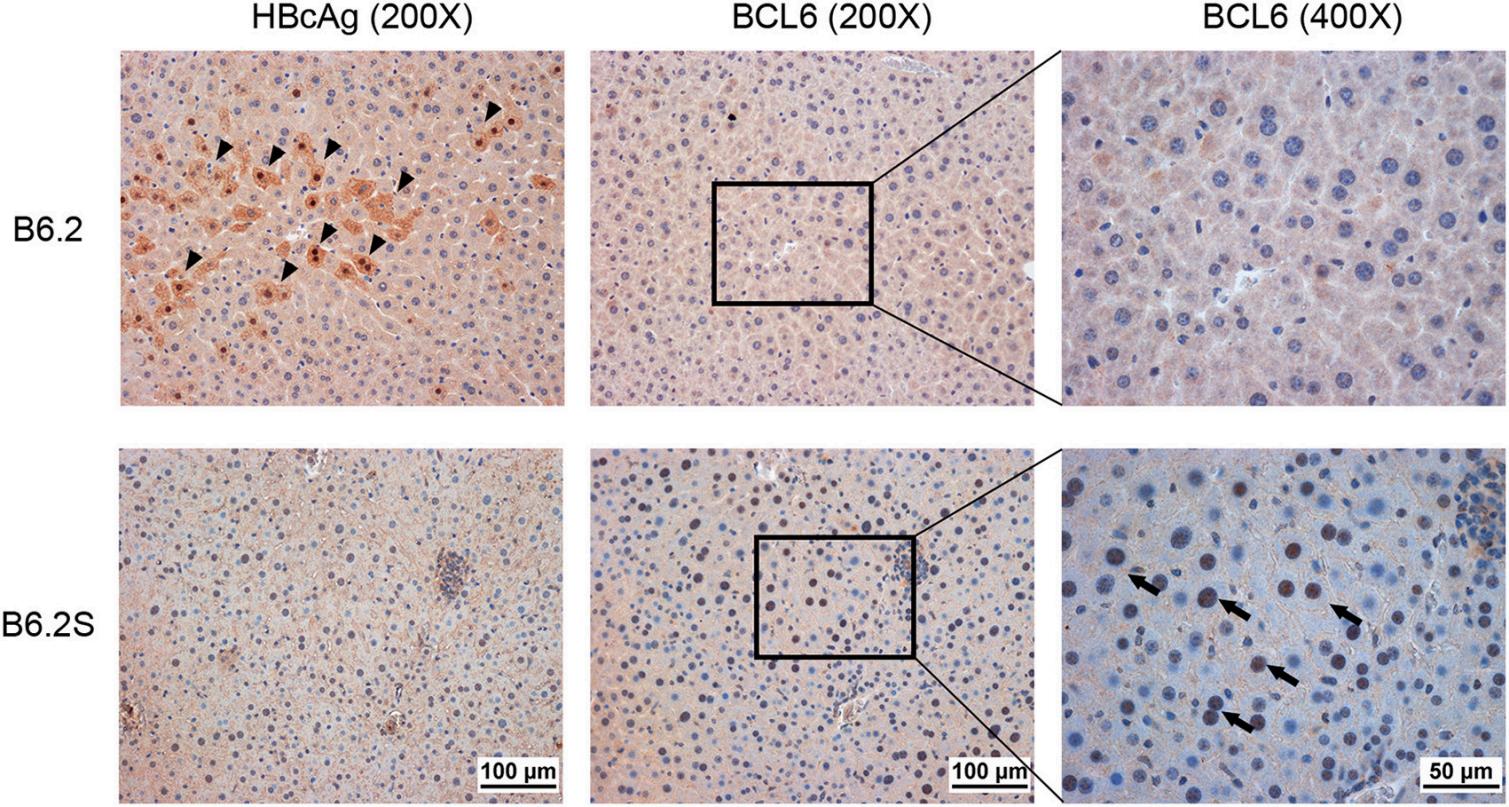
BCL6 is mainly expressed in hepatocyte nuclei in HBV-cleared mice. The liver was harvested from B6.2S mice during the HBV clearance stage and from B6.2 mice at equivalent time points. IHC staining was performed on 5-μm paraffin sections using anti-HBcAg or anti-BCL6 antibody. Arrowheads indicate the HBcAg (+) cells and arrows indicate the BCL6 (+) cells.

To verify the association of BCL6 with HBV clearance, we first examined the expression profile of *Bcl6* in the B6.2- and B6.2S-injected mouse livers. The results showed that the BCL6 levels were not significantly different between these two groups of mice at early times post-DNA injection, but started to increase in the B6.2S group from 6 to 8 w.p.i. (Figure 2C), the time that HBV was being or about to be cleared. To confirm this point in another experimental set, we harvested the B6.2S mouse liver at the time points when the serum HBeAg levels reached a COI of 1–20, representing the HBV clearance stage (*N* = 8), and B6.2 mouse livers were harvested at equivalent time points to serve as controls. The results of RT-qPCR analysis of liver RNA revealed that *Bcl6* expression was significantly higher in the B6.2S mouse liver than in the B6.2 mouse liver (Figure 2D). Immunohistochemical staining further revealed that BCL6 was abundantly produced, mainly in the nuclei of B6.2S mouse liver hepatocytes, but at relatively low levels in the B6.2 mouse liver (Figure 3). Conversely, the levels of HBV core antigen (HBcAg) remained high in the B6.2 mouse liver, but were greatly reduced in the B6.2S mouse liver, consistent with their HBV persistence or clearance status, respectively. Collectively, these results demonstrated that *Bcl6* expression was significantly upregulated in B6.2S mouse hepatocytes upon HBV clearance.

### BCL6 Overexpression Promotes HBV Clearance and Suppresses HBV Gene Expression *in vivo* and *in vitro*

**Figure 4:**
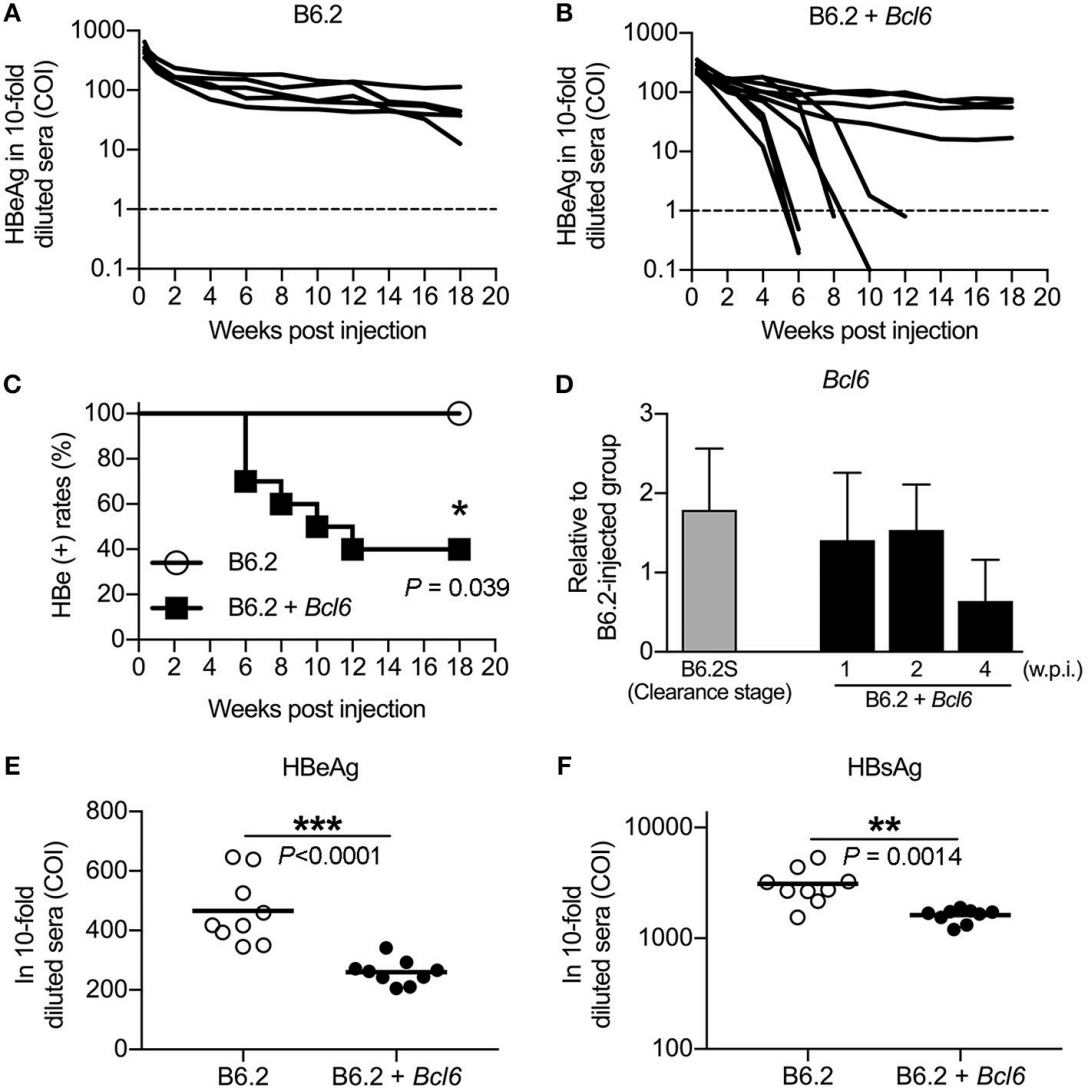
BCL6 promotes the clearance of a co-expressed persistent HBV clone and downregulates the expression of HBeAg and HBsAg *in vivo*. Mice were hydrodynamically injected with **(A)** 10 μg of B6.2 DNA and 10 μg of empty vector DNA or **(B)** 10 μg of B6.2 DNA and 10 μg of the *Bcl6*-expressing plasmid. Serum levels of HBeAg (COI) were monitored. Each line represents one mouse; the dotted lines indicate the cut-off value for HBeAg. **(C)** The kinetics of HBeAg clearance were monitored over a period of 18 weeks post-DNA injection. *P < 0.05 for the log-rank test (*N* = 5 for the B6.2 group and *N* = 10 for the B6.2 + *Bcl6* group). **(D)** Liver expression of *Bcl6* in the B6.2 + *Bcl6* mice was examined by RT-qPCR analysis on weeks 1, 2, and 4 post-DNA injection and compared with that of B6.2S mice during the HBV clearance stage (*N* = 5 each). **(E,F)** The 10-fold diluted serum levels of HBeAg and HBsAg, respectively, were measured on day 2 post-DNA injection. Each dot represents one COI value for the mouse. **P < 0.01 and ***P < 0.001 by the Student's *t*-test.

To determine whether BCL6 played a role in HBV clearance, we co-injected a *Bcl6*-expressing plasmid together with B6.2 replicon DNA, the persistent clone, into the liver of FVB/N mice, and HBV persistence was monitored. As anticipated, the B6.2 replicon alone remained persistent in the mouse liver for up to 20 weeks (Figure 4A). However, co-expression of *Bcl6* and the B6.2 replicon significantly reduced the persistence rate of B6.2 (Figures 4B,C), indicating that *Bcl6* expression could promote HBV clearance. It is noteworthy that the expression levels of *Bcl6* from the injected plasmid were comparable to those in the B6.2S mouse liver during the HBV clearance stage (Figure 4D), arguing against that HBV clearance in the (B6.2 + *Bcl6*) group was an artifact caused by *Bcl6* overexpression. Moreover, we noted that the serum levels of HBeAg (Figure 4E) and HBsAg (Figure 4F) were significantly reduced in the (B6.2 + *Bcl6*) group compared to the B6.2 group on day 2 post-DNA injection, suggesting that *Bcl6* expression might downregulate HBV gene expression in the liver.

**Figure 5:**
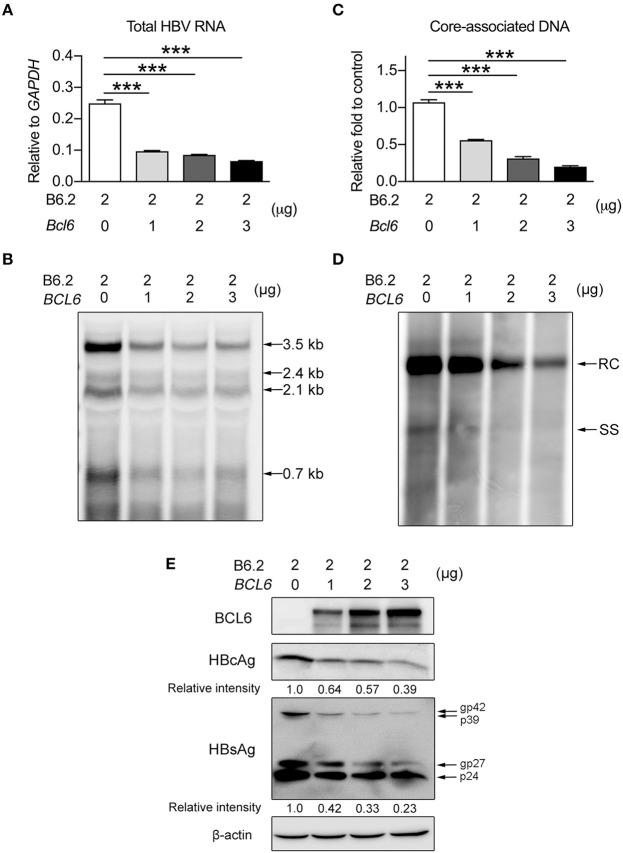
BCL6 suppresses HBV gene expression *in vitro*. Two micrograms of B6.2 replicon DNA were co-transfected with increasing amounts of the *BCL6*-expressing plasmid into Huh-7 cells. The total transfected DNA amounts were adjusted to the same using control vector DNA. Six days later, the viral RNA levels were measured **(A)** by RT-qPCR analysis using a pair of primers residing in the X region, or **(B)** by Northern blot analysis using a probe in the X region. The intracellular core-associated viral DNA was examined by **(C)** qPCR or **(D)** Southern blot analysis using the same primers and probe, respectively. **(E)** The protein levels were analyzed by Western blot using anti-HBcAg, anti-HBsAg, anti-BCL6 and anti-β-actin antibodies. The data presented in **(A,C)** are the mean ± *SD* of three independent experiments. ***P < 0.001 for one way-ANOVA followed by Dunnett's test. RC, relaxed circular HBV DNA; SS, single-stranded HBV DNA; and gp, glycoprotein.

To verify this phenomenon *in vitro*, we co-transfected Huh-7 cells with the B6.2 replicon DNA and the *BCL6*-expressing plasmid and examined HBV DNA expression and replication. A pair of PCR primers residing in the HBV X region were used to detect total HBV RNA levels. The results showed that *BCL6* expression reduced total HBV RNA levels in a dose-dependent manner (Figure 5A) and all four viral RNA species (3.5, 2.4, 2.1, and 0.7 kb) were impacted, as shown by Northern blot analysis (Figure 5B). Consequently, the levels of the downstream products of the HBV replication cycle, i.e., the intracellular core-associated viral DNAs, detected by qPCR (Figure 5C) or by Southern blot analysis (Figure 5D), and the viral proteins (Figure 5E) were all correspondingly reduced. Altogether, the *in vivo* and *in vitro* results indicated that BCL6 might suppress HBV gene expression.

### BCL6 Suppresses HBV Gene Expression Independent of HBV Genotype and Also in HepG2 Cells

**Figure 6:**
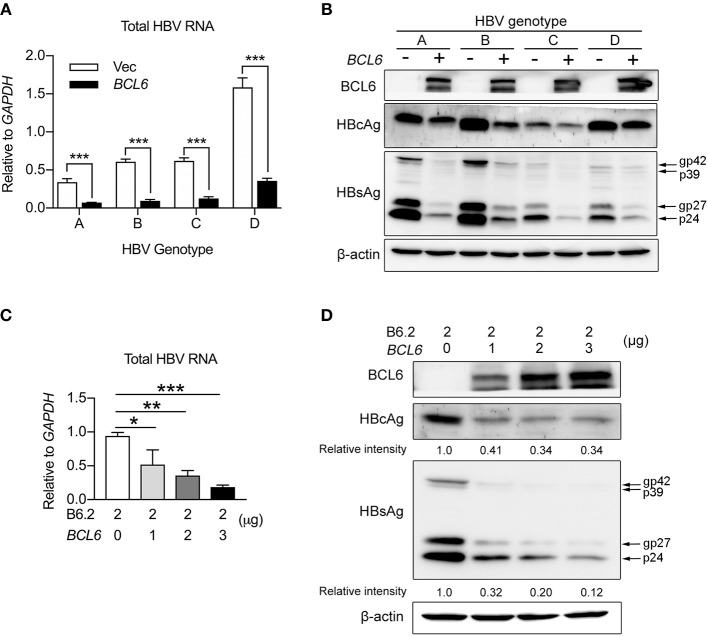
BCL6 suppresses HBV gene expression independent of HBV genotype and also in HepG2 cells. **(A,B)** Huh-7 cells were co-transfected with 2 μg of HBV replicon DNA of genotypes **(A–D)** together with 3 μg of the *BCL6*-expressing plasmid. **(C,D)** HepG2 cells were co-transfected with 2 μg of B6.2 replicon DNA and increasing amounts of the *BCL6*-expressing plasmid. Six days later, the viral RNA levels **(A,C)**, and viral proteins **(B,D)**, were examined by RT-qPCR and Western blot, respectively. The data presented in **(A,C)** are the mean ± *SD* of three independent experiments. In **(A)**, ***P < 0.001 with the Student's *t*-test. In **(C)**, *P < 0.05 and **P < 0.01 with one way-ANOVA followed by Dunnett's test.

Next, we examined whether the inhibitory effect of BCL6 on HBV gene expression was a universal phenomenon. HBV replicon DNAs from different HBV genotypes (A, B, C, and D) were co-transfected with the *BCL6*-expressing plasmid into Huh-7 cells, or B6.2 replicon DNA and the *BCL6*-expressing plasmid were co-transfected into another hepatoma cell line, HepG2. The results showed that *BCL6* expression significantly reduced the RNA (Figure 6A) and protein levels (Figure 6B) of HBV replicons from all four genotypes. Similarly, the levels of B6.2 viral RNA (Figure 6C) and viral proteins (Figure 6D) all dose-dependently decreased with the increase of BCL6 in HepG2 cells. These results demonstrated that the inhibitory effects of BCL6 on HBV gene expression were independent of HBV genotype and also effective in other hepatoma cells.

### BCL6 Functions as a Transcriptional Repressor of HBV Promoters

**Figure 7:**
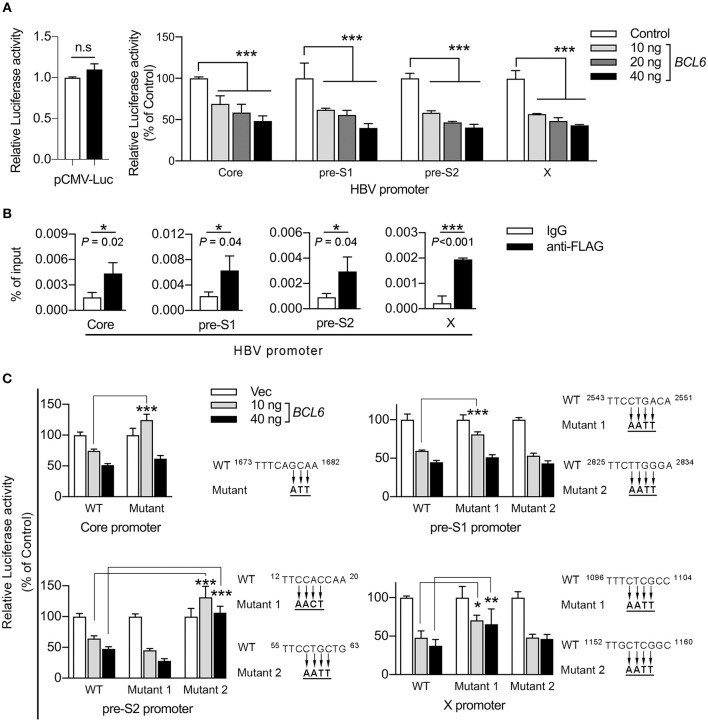
BCL6 functions as a transcriptional repressor by binding to the HBV promoters. **(A)** The luciferase gene was constructed under the control of the CMV promoter or one of the four HBV promoters, including core, pre-S1, pre-S2, and X. Huh-7 cells were co-transfected with the reporter plasmid together with increasing amounts of the *BCL6*-expressing plasmid. The CMV-RL plasmid (*Renilla* luciferase) was also co-transfected to normalize the transfectional efficiency. Luciferase activity was measured 2 days after DNA transfection. **(B)** ChIP assay was performed to confirm the binding of BCL6 to HBV promoters. Huh-7 cells were co-transfected with B6.2 replicon DNA and the *Flag-BCL6*-expressing plasmid. Two days later, the BCL6-bound chromatin was immunoprecipitated with anti-Flag antibody or control IgG. The DNA fragments were eluted and then subjected to qPCR analysis using primers from the four HBV promoter regions. The vertical axis indicates the relative intensities of PCR products normalized to input DNA. **(C)** The putative BCL6 binding sites in each HBV promoter were mutated as indicated, and the luciferase gene was constructed under the control of the mutant promoters. The luciferase activity assays were conducted as described in **(A)**. All the data presented are the mean ± *SD* of three independent experiments. *P < 0.05, **P < 0.01, ***P < 0.001, and n.s., not significant. In **(A)**, one way-ANOVA followed by Dunnett's test; in **(B)**, the Student's *t*-test and in **(C)**, two way-ANOVA followed by Bonferroni's test were used.

To determine whether BCL6 downregulated HBV gene expression at the transcriptional level, we performed reporter assays in Huh-7 cells in which the firefly luciferase gene was under the control of individual HBV promoters, i.e., the core, pre-S1, pre-S2, and X promoters, or a control cytomegalovirus (CMV) promoter. The results showed that *BCL6* expression did not influence CMV promoter activity, but attenuated the activities of all four HBV promoters in a dose-dependent manner (Figure 7A).

BCL6 has been reported to bind to a major consensus motif, TTCCT(A/G)(G/A)A(A/G), in various promoters to suppress gene expression in GC B cells (Basso et al., 2010). Using the FIMO (Find Individual Motif Occurrences) program (Grant et al., 2011), we identified 1–2 putative binding sites in each of the HBV promoters and the proximal downstream regions (Supplementary Figures S1–S4). Next, we performed chromatin immunoprecipitation (ChIP) assays to investigate whether BCL6 could bind to HBV promoters or the proximal downstream regions. Huh-7 cells were co-transfected with B6.2 replicon DNA and the *Flag-BCL6*-expressing plasmid. Anti-Flag antibody was used to pull down the BCL6-bound DNA fragments which were further analyzed by qPCR. Our results showed that significantly more DNA fragments were immunoprecipitated from all four HBV promoters by anti-Flag antibody than by control IgG (Figure 7B), suggesting that BCL6 could bind to HBV promoters or the proximal downstream regions.

To correlate this binding with the inhibitory effects of BCL6 on HBV promoter activities, we further mutated the putative BCL6-binding sites in each promoter (Figure 7C) and repeated the reporter assays. The results shown in Figure 7C indicated that mutation on the putative binding site of the core promoter, or on the first but not the second site of the pre-S1 promoter, significantly reduced the BCL6 inhibition on these promoters at low concentration (10 ng) but not at high concentration (40 ng) of the repressor. We speculated that these mutations might be insufficient to completely preclude BCL6 binding to these mutation sites, especially at high concentration of the repressor. Mutation on the second site of the pre-S2 promoter or the first site of the X promoter also significantly reversed the inhibitory effects of BCL6 at either low or high concentration. Notably, the mutations in the pre-S1 and the X promoters did not fully reverse the BCL6 inhibition, which could be due to the insufficient mutations on these sites or due to the presence of other binding sites residing elsewhere in the promoter region, allowing BCL6 to partially bind to the mutant promoter. Nevertheless, these results indicated that BCL6 could bind to and function as a transcriptional repressor for all four HBV promoters.

### BCL6 Also Functions as an Immune Modulator to Augment Immune Cell Infiltration Into the Liver

**Figure 8:**
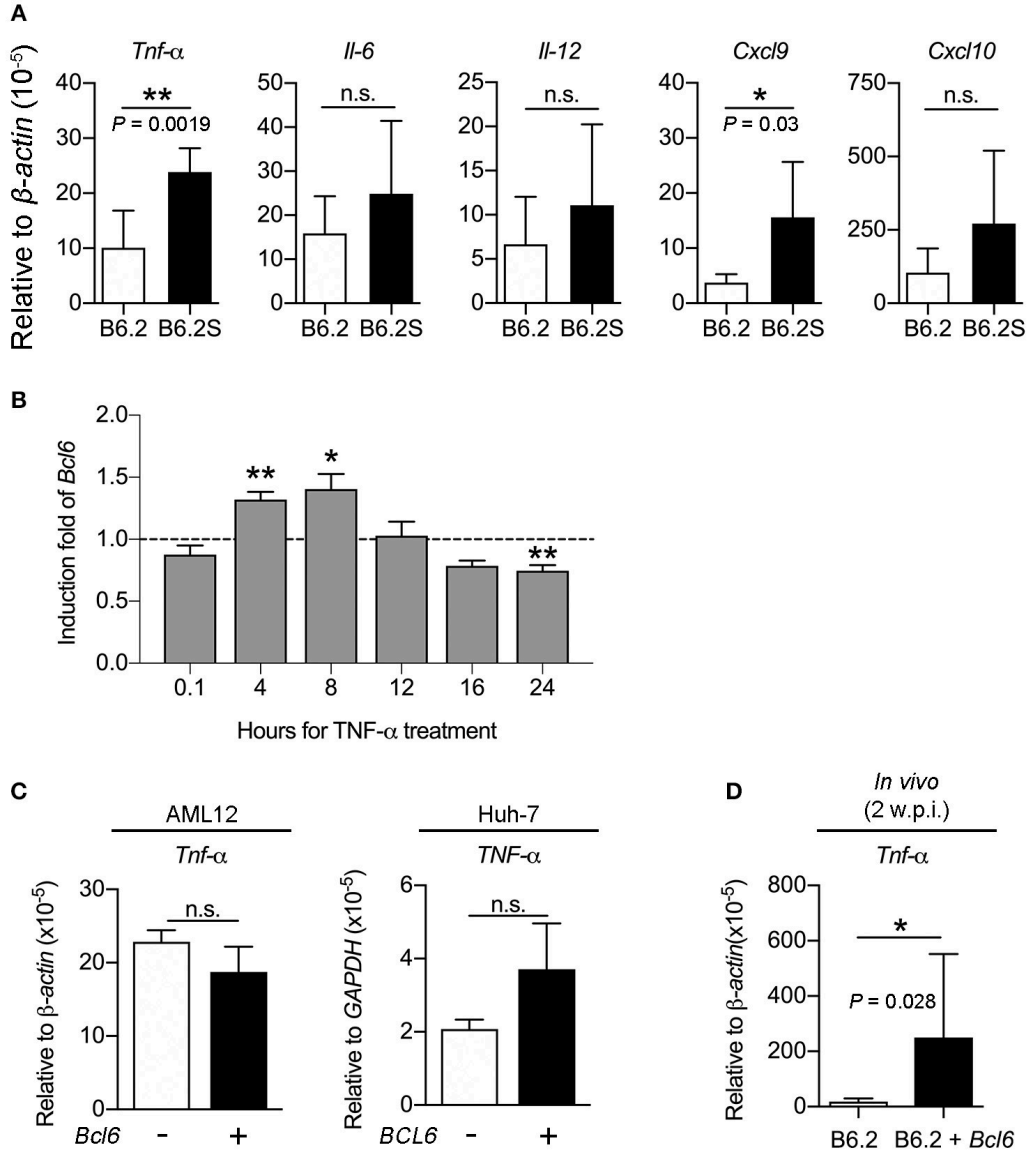
TNF-α induces *Bcl6* gene expression in the hepatocytes and *Bcl6* overexpression upregulates the TNF-α levels *in vivo*. **(A)** The RNA levels of *Tnf-*α, *Il-6, Il-12, Cxcl9, and Cxcl10* were examined by RT-qPCR in B6.2- and B6.2S-injected mouse liver isolated during the HBV clearance stage. **(B)** AML12 cells were treated with or without TNF-α (100 ng/ml) for the indicated time periods and *Bcl6* RNA was analyzed by RT-qPCR. The induction fold means the ratio of the *Bcl6* RNA levels in the presence of TNF-α over those without TNF-α. **(C)** AML12 and Huh-7 cells were transfected with 1 μg of vector or the *Bcl6*-expressing plasmid. Two days later, the TNF-α RNA levels were analyzed by RT-qPCR analysis. **(D)** The liver RNA from the B6.2 or the (B6.2 + *Bcl6*) mice at 2 w.p.i. were analyzed for *Tnf-*α expression by RT-qPCR. In **(A–D)**, *P < 0.05, **P < 0.01, n.s., not significant with the Student's *t*-test.

It has been reported that BCL6 expression could be stimulated by several cytokines, including IFN-γ, IL-6, type I IFN, IL-12, and TNF-α in various cell types (Hideshima et al., 2010; Nakayamada et al., 2011, 2014; Choi et al., 2013; Madapura et al., 2017; Ujvari et al., 2018). To verify whether the upregulation of BCL6 in the B6.2S mouse liver resulted from increased levels of cytokines upon HBV clearance, we first compared the expression levels of these cytokines in the liver of B6.2 and B6.2S mice. The results showed that TNF-α, IL-6, and IL-12 were expressed at higher levels in the B6.2S mouse liver during the clearance stage than those in the B6.2 mouse liver, but only the difference of TNF-α levels reached statistical significance (Figure 8A); by contrast, the levels of IFN-β and IFN-γ were too low to be detected in both groups (data not shown). Concurrently, the CXCL9 and CXCL10 levels were also significantly upregulated in the B6.2S mouse liver (Figure 8A). We next demonstrated that *Bcl6* expression in an immortalized mouse hepatocyte cell line, AML12, could be upregulated by TNF-α at 4–8 h post-treatment (Figure 8B), supporting the possibility that the elevated levels of BCL6 in the B6.2S mouse liver might be stimulated by the increased levels of TNF-α. On the other hand, we examined whether or not BCL6 upregulation could lead to more TNF-α induction. The results shown in Figure 8C indicated that the TNF-α levels were similar in the AML12 cells or the Huh-7 cells with or without *Bcl6* overexpression. In contrast, *Bcl6* overexpression in the (B6.2 + *Bcl6*)-injected mice significantly upregulated the TNF-α levels in the livers (Figure 8D), suggesting that the intrahepatic upregulation of *Bcl6* could lead to more TNF-α production from the cells in the environment.

**Figure 9:**
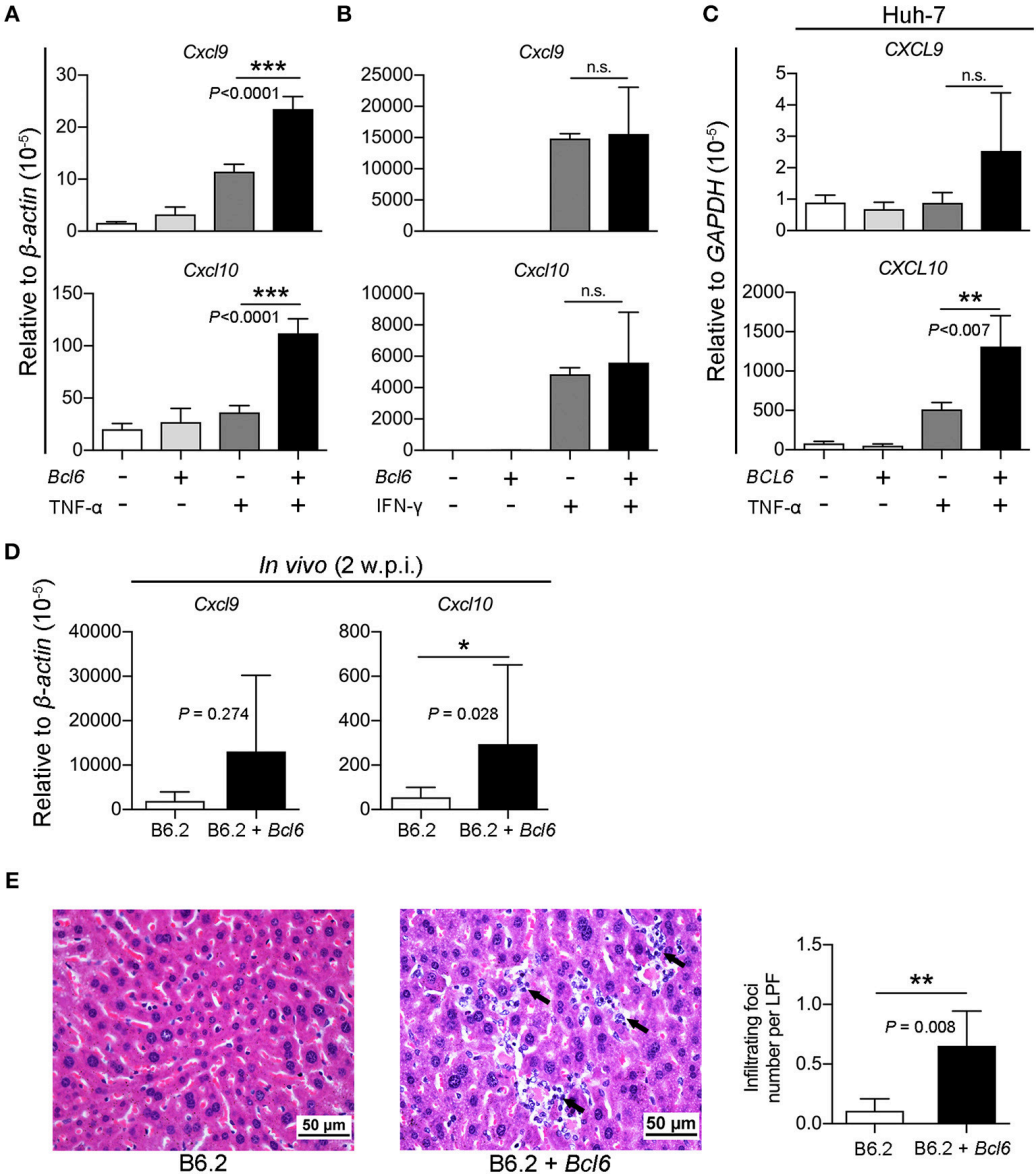
BCL6 expression synergizes TNF-α-induced *Cxcl9/Cxcl10* expression and enhances immune cell infiltration into the liver. **(A,B)** AML12 cells, **(C)** Huh-7 cells, were transfected with 1 μg of vector or the *Bcl6*-expressing plasmid. Two days later, the cells were treated with or without **(A,C)** TNF-α (100 ng/ml) or **(B)** IFN-γ (50 ng/ml) for 3 h. RNA was extracted and analyzed for *Cxcl9/Cxcl10* expression by RT-qPCR. **(D)** The liver RNA from the B6.2 or the (B6.2 + *Bcl6*) mice at 2 w.p.i. were analyzed for *Cxcl9/Cxcl10* expression by RT-qPCR and **(E)** the liver tissues were analyzed by hematoxylin & eosin staining. The arrows indicate the infiltrating immune cells. The images are displayed at 400X magnification. The average number of infiltrating foci were obtained from 15 different fields for each mouse (*N* = 5) at 200X magnification and presented as the mean ± *SD*. LPF, low power field. *P < 0.05, **P < 0.01, and ***P < 0.001. In **(A–C)**, one way-ANOVA followed by Dunnett's test, and in **(D,F)**, the Student's *t*-test were used.

Thus, we investigated whether the intrahepatic expression of *Bcl6* might impact cytokine signaling in the cells. The expression of *Cxcl9* and *Cxcl10* in response to TNF-α or IFN-γ (as a control) stimulation was examined in the AML12 cell line in the presence or absence of *Bcl6* expression. Interestingly, the results showed that *Bcl6* alone did not induce *Cxcl9*/*Cxcl10* expression, but it markedly augmented *Cxcl9*/*Cxcl10* expression stimulated by TNF-α (Figure 9A) but not by IFN-γ (Figure 9B). Similar phenomena were also observed in the Huh-7 cells expressing *BCL6* and stimulated with TNF-α (Figure 9C). In corroboration with the *in vitro* findings, ectopic expression of *Bcl6 in vivo* also significantly increased *Cxcl9*/*Cxcl10* expression in the liver (Figure 9D), albeit *in vivo Cxcl9*/*Cxcl10* expression might not be induced by TNF-α alone. Importantly, the increased levels of CXCL9/CXCL10 in the (B6.2 + *Bcl6*)-injected mice resulted in significantly higher levels of infiltrating immune cells in their livers (Figure 9E). Taken together, our results suggested that higher levels of cytokines, especially TNF-α, in the B6.2S mouse liver might stimulate intrahepatic expression of *Bcl6*, which in turn, synergizes TNF-α signaling to produce high levels of chemokines, such as CXCL9 and CXCL10, and promote immune cell infiltration into the liver to clear HBV.

## Discussion

Clones B6.2 and B6.2S differ from each other by only one nucleotide, leading to one amino acid change (N214S) in HBsAg. This nucleotide change, however, does not change the amino acid composition of the polymerase. We have previously demonstrated that the *in vitro* replication efficiencies of B6.2 and B6.2S are comparable (Chen et al., 2012). Thus, the opposed persistence rates of these two clones might result from the differences in the in *vivo* responses, but not the replication efficiency. Taking advantage of the HBV persistent model established in the inbred FVB/N mice and the minimal sequence variation between the B6.2 and B6.2S clones, thus reducing the undesired heterogeneity of immune responses, this study compared the immune-related host factors that were differentially expressed in these two groups of animals, which might be associated with HBV clearance. BCL6 was identified to be significantly upregulated in the B6.2S-injected mouse liver at the time when HBV was being cleared. Although the increment of BCL6 in the B6.2S mice was only about 2-fold in comparing with the B6.2 mice based on RT-qPCR analysis (Figure 2D), it is noteworthy that the RNA was extracted from total liver, in which only a small fraction of hepatocytes (probably < 10%) were transfected with HBV DNA due to the low HD efficiency, and thus the induction fold of BCL6 RNA was greatly diluted by the RNA of untransfected cells. According to this scenario, we predicted that the local concentration of BCL6 in the B6.2S DNA-injected loci would be more than 2-fold as compared to that in the B6.2 DNA-injected loci. The prediction was actually confirmed by the IHC staining result of HBV DNA-injected livers, which showed scattered patterns of BCL6-expressing loci in the liver but in each locus many cells were stained positive for BCL6 (Figure 3). Our studies also demonstrated that BCL6 suppressed the activities of HBV promoters *in vitro* and *in vivo* and that *Bcl6* expression could be stimulated by inflammatory cytokines such as TNF-α; the BCL6 in turn synergized TNF-α signaling to produce large amounts of CXCL9 and CXCL10, leading to high levels of immune cells infiltrating the liver. These immune cells might further produce high levels of cytokines such as TNF-α, IL-6, IL-12, and so on, resulting in positive feedback loops on *Bcl6* expression and immune responses, which might ultimately lead to HBV clearance. Most importantly, the result demonstrating that co-expression of *Bcl6*, at levels similar to those in the B6.2S mice, and the persistent clone B6.2 significantly accelerated the clearance of HBeAg in B6.2 mice strongly argues the important role of BCL6 in immune control of HBV infection. Thus, BCL6 represents a novel host restriction factor that can be induced under inflammatory conditions and exerts both anti-HBV and immunomodulatory activities.

BCL6 is a well-known transcriptional repressor that exerts its function either by directly recruiting histone deacetylase complexes (HDAC) or through co-repressors (Basso and Dalla-Favera, 2010). Interestingly, the BCL6 consensus binding motif was found in the promoter or in nearby regions in all four HBV promoters, which was important for the inhibitory effects of BCL6 on HBV promoter activities, suggesting that BCL6 might act as a direct transcriptional repressor on these promoters. The binding motifs were conserved in the promoter regions of other HBV genotypes (Supplementary Figures S1–S4), explaining why the expression of other HBV genotypes was also suppressed by BCL6.

BCL6 expression can be stimulated by cytokines such as IFN-γ, IL-6, type I IFN, IL-12, and TNF-α in myeloma or T_FH_ cells (Hideshima et al., 2010; Nakayamada et al., 2011, 2014; Choi et al., 2013; Madapura et al., 2017; Ujvari et al., 2018). In our previous (Chen et al., 2012) and current studies, we have found significantly higher levels of TNF-α in the B6.2S mouse liver than in the B6.2 mouse liver during the HBV clearance stage. TNF-α is a cytokine produced by activated cytotoxic T lymphocytes (CTLs) or antigen-non-specific macrophages and T cells, and it can non-cytolytically suppress HBV replication in transgenic mouse models (Guidotti and Chisari, 1996, 1999). These effects can now be attributed to several unique mechanisms. First, TNF-α inhibits HBV replication by blocking the formation or stability of HBV core particles (Biermer et al., 2003; Puro and Schneider, 2007). Second, TNF-α can reduce HBV cccDNA levels in the hepatocytes by inducing cccDNA deamination and degradation through the upregulation of the cytidine deaminase APOBEC3B (Xia et al., 2016). Third, TNF-α can inhibit HBV core promoter activity, but the mechanism was unclear (Romero and Lavine, 1996; Uprichard et al., 2003). Our findings demonstrating that TNF-α can stimulate *Bcl6* expression, which in turn inhibits HBV gene expression, thus add a new function for TNF-α in the non-cytolytic mechanism of HBV clearance.

An interesting question then arises as to why expressing B6.2S DNA would induce higher levels of TNF-α than expressing B6.2 DNA *in vivo*. Here, we provide two, but maybe not the last, possibilities to explain the difference. First, expressing B6.2S DNA induced higher levels of CTL activity than expressing B6.2 DNA in animals, as shown by our previous study (Chen et al., 2012). The increased CTLs led to an early increase in intrahepatic IFN-γ, TNF-α, CXCL9, and CXCL10 levels in B6.2S mice (Chen et al., 2012). We have recently identified a CTL epitope site (aa 202–aa 213) in the HBsAg region, which is right next to the mutation site (N214S). Thus, we speculate that the Ser-214 residue may lead to the more efficient processing of HBsAg in antigen-presenting cells than the Asn-214 residue, thus activating higher CTL activity and producing higher levels of cytokines for the B6.2S clone. The initiative CTLs may be prerequisite for the subsequent amplification of antigen-non-specific inflammatory cells and elevated levels of TNF-α essential for HBV clearance. We are currently working on this issue. Second, we have unexpectedly found that animals injected with B6.2S DNA secreted much lower levels of HBsAg into the sera compared to those injected with B6.2 DNA (Supplementary Figure S5). We speculated that the poor secretion might be due to the N214S mutation in the HBsAg. Since HBsAg can antagonize innate immunity in several ways (Wu et al., 2009; Xu et al., 2009; Shi et al., 2012), it is predicted that the B6.2S clone may induce lower antagonism against host immune responses. Hence, more immune cells can be activated and higher levels of cytokines such as TNF-α are produced in the liver upon injection with B6.2S DNA than with B6.2 DNA.

As a transcriptional repressor, BCL6 has been reported to antagonize the activities of NF-κB-stimulated promoters (Barish et al., 2010). Surprisingly, this study found that TNF-α-induced *Cxcl9/Cxcl10* expression, which also depends on NF-κB activity, was actually enhanced by BCL6. However, unlike the study by Xu et al., which reported that BCL6 activated NF-κB activity *via* restraining *Irf7* transcription in VSV-infected RAW264.7 cells (Xu et al., 2016), we found that *Bcl6* expression did not influence NF-κB activity or *Irf7* expression in the AML12 cell line (data not shown), indicating that the BCL6 stimulation of *Cxcl9/Cxcl10* expression in hepatocytes was independent of NF-κB signaling. It remains to be determined how BCL6 enhances TNF-α signaling to increase CXCL9/CXCL10 production in the hepatocytes.

**Figure 10:**
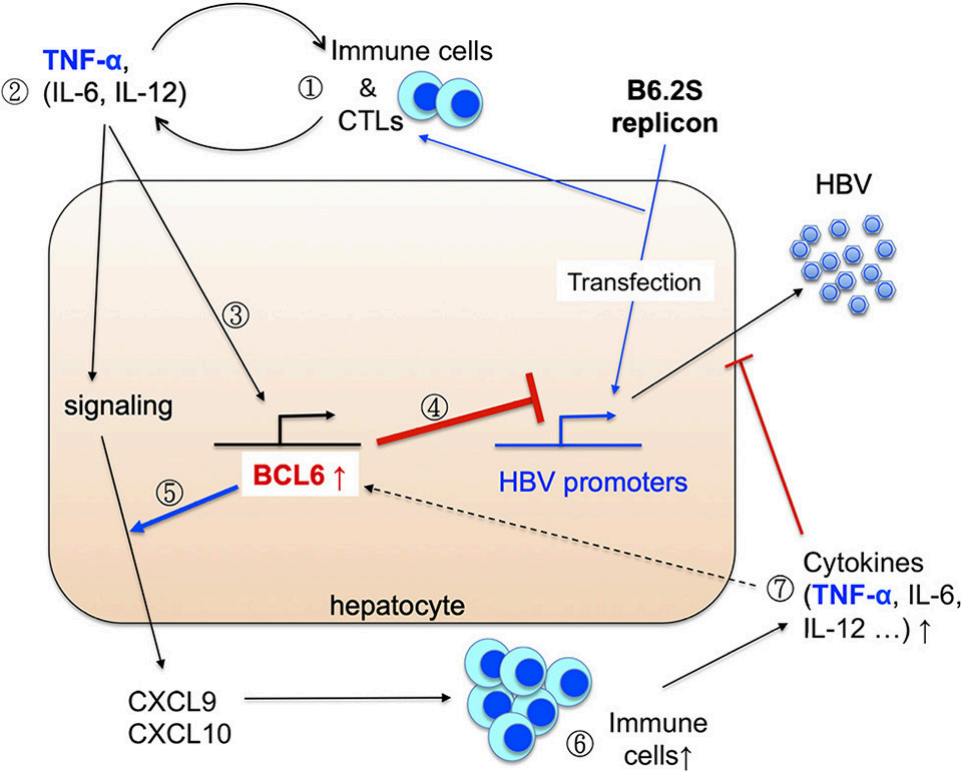
Working model for the induction and anti-HBV activity of BCL6 *in vivo*. Please see Discussion for detail. We propose that (1) the initiative CTLs may be generated in the livers expressing the B6.2S replicon DNA (Chen et al., 2012), followed by infiltration of many other immune cells. (2) These immune cells produce diverse cytokines in the liver. (3) The inflammatory cytokines such as TNF-α can upregulate *Bcl6* expression. (4) BCL6 can repress HBV promoter activity, and (5) meanwhile synergizes TNF-α signaling to produce large amounts of CXCL9/CXCL10 chemokines. (6) The chemokines recruit more immune cells infiltrating to the livers and (7) produce higher levels of cytokines and chemokines. These immune cells, cytokines together with the BCL6 functions may ultimately lead to HBV clearance.

Based on our previous and current findings, we thus propose a working model to reveal how BCL6 is induced in the B6.2S mice to exert its anti-HBV activities *in vivo* (Figure 10). In the B6.2S DNA-injected mice, CTLs may be activated in the beginning due to the unique Ser-214 amino acid present in the HBsAg region, leading to initial elevation of inflammatory cytokines and chemokines in the liver, which subsequently recruit antigen-non-specific inflammatory immune cells infiltrating the DNA-injected liver and secreting more proinflammatory cytokines. Among them, TNF-α, and possibly IL-6 and IL-12 as well (Hideshima et al., 2010; Nakayamada et al., 2011; Choi et al., 2013), can upregulate BCL6 expression in the hepatocytes. The BCL6 protein not only directly represses HBV gene expression, but also synergizes TNF-α signaling to produce large amounts of CXCL9/CXCL10 chemokines, which in turn recruit even more immune cells into the liver and produce high levels of BCL6 and cytokines that can block HBV replication. The positive feedback loops between BCL6 expression and immune responses may ultimately lead to a complete clearance of HBV. These effects point to novel roles for BCL6 as a host restriction factor and offer a new potential intervention avenue against HBV infection. To establish biological relevance of this work, we may need to further demonstrate the BCL6 regulation of cccDNA gene expression in HBV infection system and the TNF-α induction of BCL6 expression in human hepatocytes during the remission stage of acute HBV infection in the future. The original gel blots for all of the figures are shown in Supplementary Figures S6, S7.

## Author Contributions

L-HH conceived the project. L-HH and C-TL wrote the manuscript. C-TL, Y-TH, Y-JY, S-HC, and C-HW conducted the experiments.

### Conflict of Interest Statement

The authors declare that the research was conducted in the absence of any commercial or financial relationships that could be construed as a potential conflict of interest.
