# Supplementary material for: B-Cell Lymphoma 6 (BCL6) Is a Host Restriction Factor That Can Suppress HBV Gene Expression and Modulate Immune Responses
Source: Front Microbiol. 2019 Jan 10;9:3253. doi: 10.3389/fmicb.2018.03253 (PMC6335256; doi:10.3389/fmicb.2018.03253)
Supplement: Supplementary file 1 [file Data_Sheet_1.PDF]

Supplementary Table S1: Primer sequences

**Target gene**	**Forward primer (5'-3')**	**Reverse primer (5'-3')**
Primers for qPCR		
Mouse *Bcl6*	CCCCGGCTGACAGCTGTAT	ATGACAACGTCCGTCAAGATGT
Human *BCL6*	GCCAGTCCCTGGTGAGTACAG	TGCATARCACTTCGTGCCTCTT
Il-6	TGGTGACAACCACGGCCTTCC	AAGCCTCCGACTTGTGAAGTGGT
Mouse *Tnf-α*	CCTCCTGGCCAACGGCATGG	TCGGCTGACGGTGTGGGTGA
Human *TNF-α*	TCCTTCAGACACCCTCAACC	AGGCCCCAGTTTGAATTCTT
Il-12	AGAGTTTCTAGATGCTGGCCAGTAC	TTGTGGAGCAGCAGATGTGAGT
Il-17d	TTGTAGTGCTCAGATGAACTTCCTTCT	CCAGATCCGGAGCACTCATT
Il-23a	CCACAAGGACTCAAGGACAACAG	CCCCTTTGAAGATGTCAGAGTCA
Mouse *Cxcl9*	GATCAAACCTGCCTAGATCC	GGCTGTGTAGAACACAGAGT
Mouse *Cxcl10*	CGCTGAGAGACATCCCGAGC	TGCTGTCCATCACAGCACCG
Human *CXCL9*	CCACCTACAATCCTTGAAAGACCTTA	GACATGTTTGAACTCCATTCTTCAGT
Human *CXCL10*	CAATTTTGTCCACGTGTTGAGATC	CTTTTCCTTGCTAACTGCTTTCA
GAPDH	GTATTGGGCGCCTGGTCACC	CGCTCCTGGAAGATGGTGATGG
β-actin	GATCTGGCACCACACCTTCT	CTTTTCACGGTTGGCCTTAG
HBV detection	CAGCTTGGAGGCTTGAACAGT	AGGAGGCTGTAGGCATAAATTGG
HBV Core promoter	CCAAGGTCTTGCATAAGAGGA	CCAACTCCTCCCACTCAGTAAA
HBV pre-S1 promoter	TGCCCTTAGATAAAGGGATCAA	ATACCCGCCTTCCAAAGAGT
HBV pre-S2 promoter	CTCCTCATGGGGGACTGTT	GTAGGCTGCCTTCCTGACTG
HBV X promoter	TCTGAACCTTTACCCCGTTG	GAGTTCCGCAGTATGGATCG
Primers for the probe used for Southern and Northern blot		
HBx probe	CCGCTCGAGCCATGGCTGCTAGGCTGTC	CATGCCATGGTGGCAGAGGTGAAAAAGTTGC

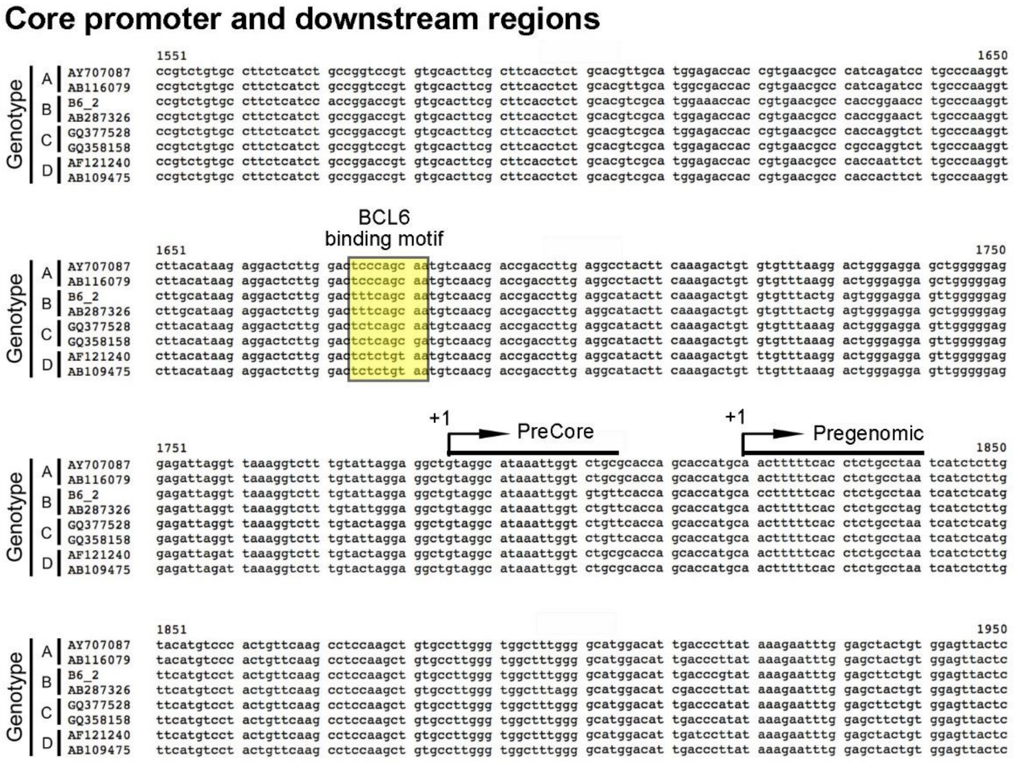

Supplementary Figure S1. The putative BCL6 binding site was found in the core promoter and downstream regions of four genotypes of HBV. The sequences in the core promoter and downstream regions (nt 1551- nt 1950) of two representative clones for HBV genotypes A, B, C and D were extracted from NCBI data base and aligned. The putative BCL6 binding sites are indicated by the yellow boxes; +1 indicates the transcription start site.

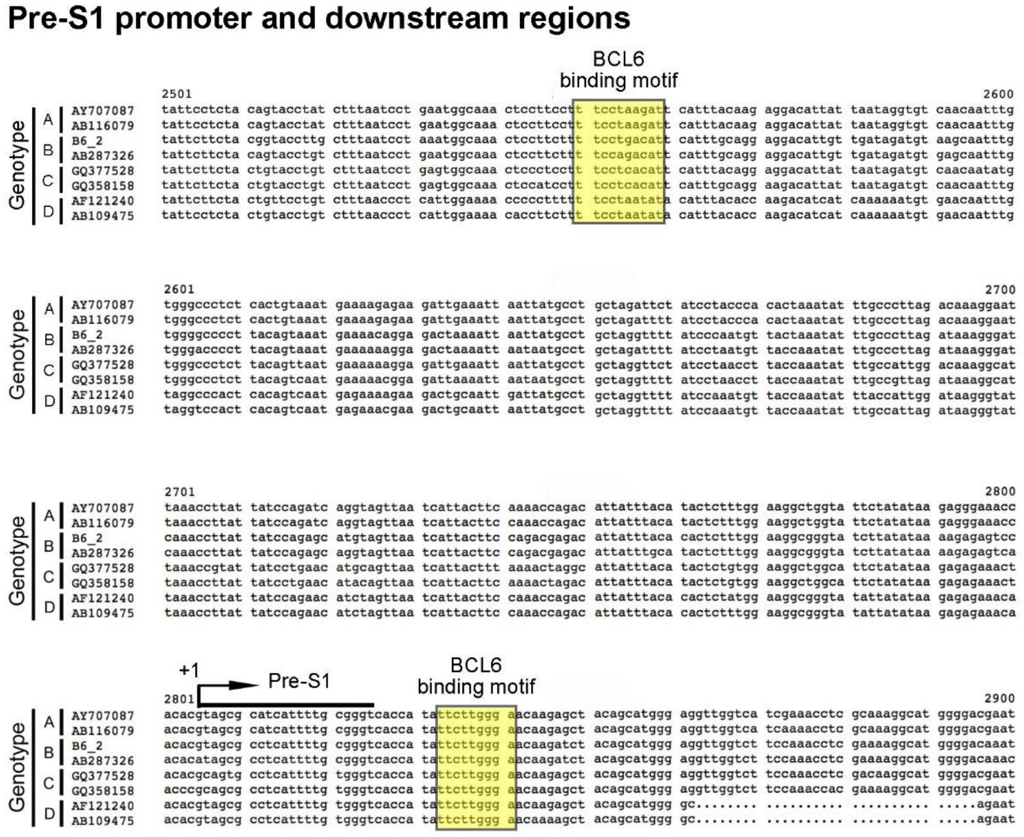

Supplementary Figure S2. The putative BCL6 binding sites were found in the pre-S1 promoter and downstream regions of four genotypes of HBV. The sequences in the pre-S1 promoter and downstream regions (nt 2501- nt 2900) of two representative clones for HBV genotypes A, B, C and D were extracted from NCBI data base and aligned. The putative BCL6 binding sites are indicated by the yellow boxes; +1 indicates the transcription start site.

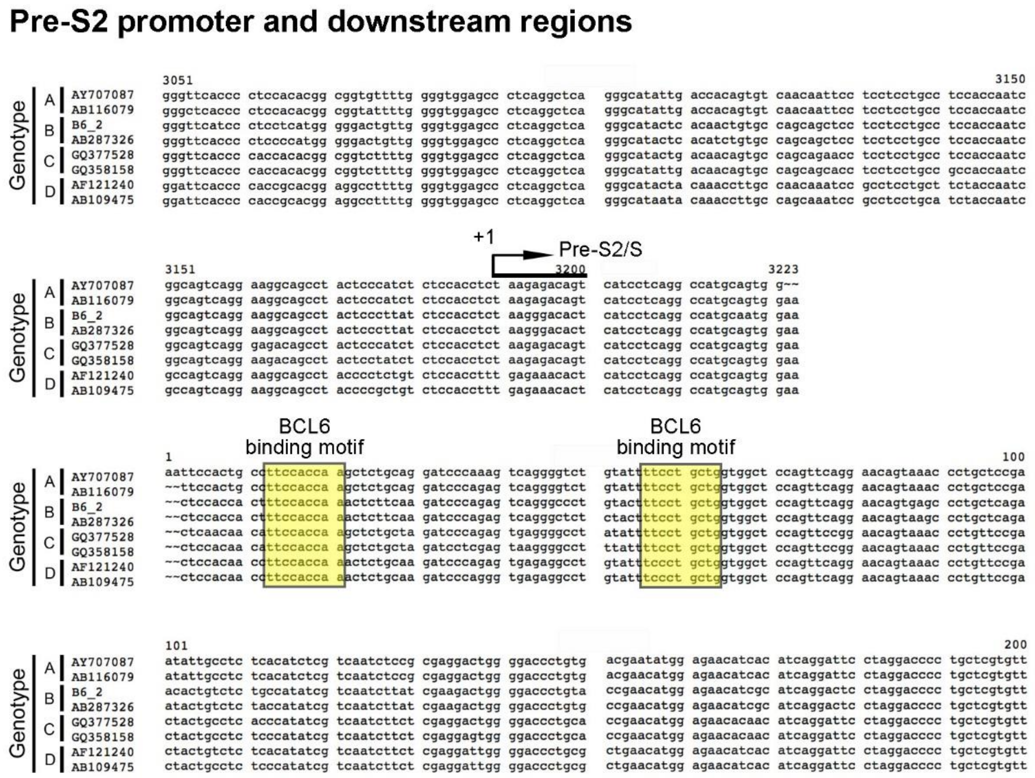

Supplementary Figure S3. The putative BCL6 binding sites were found in the pre-S2 promoter and downstream regions of four genotypes of HBV. The sequences in the pre-S2 promoter and downstream regions (nt 3051-nt 3223/1- nt 200) of two representative clones for HBV genotypes A, B, C and D were extracted from NCBI data base and aligned. The putative BCL6 binding sites are indicated by the yellow boxes; +1 indicates the transcription start site.

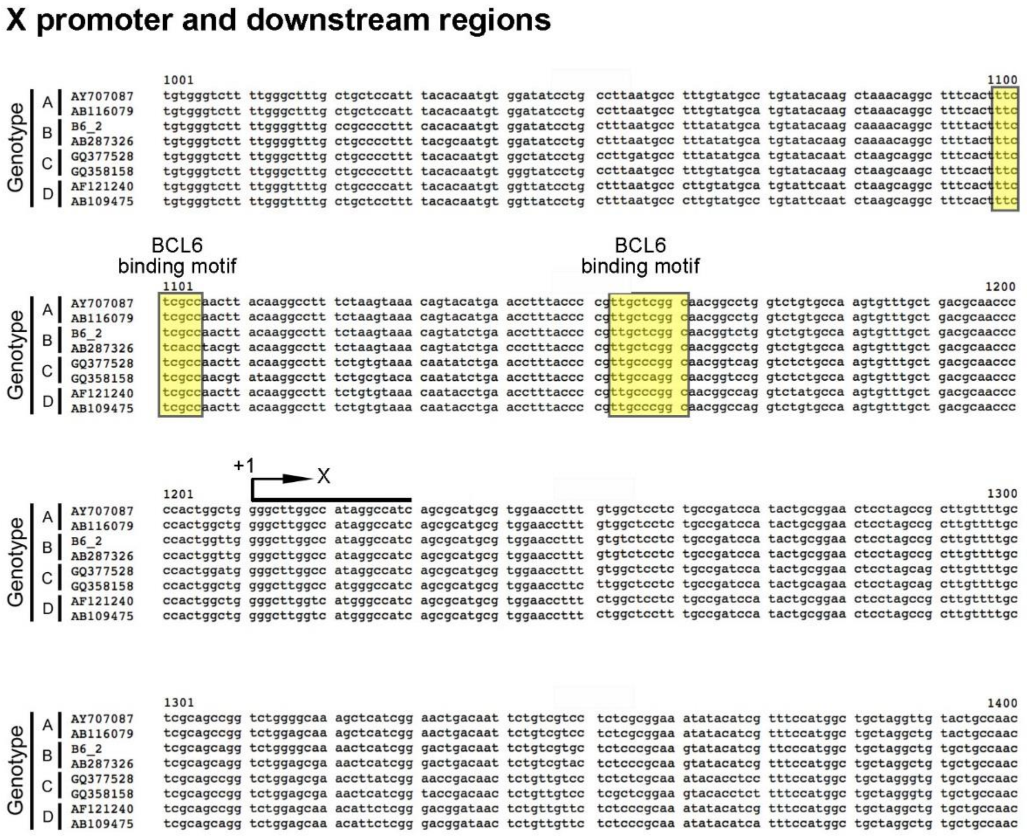

Supplementary Figure S4. The putative BCL6 binding sites were found in the X promoter and downstream regions of four genotypes of HBV. The sequences in the X promoter region (nt 1001- nt 1400) of two representative clones for HBV genotypes A, B, C and D were extracted from NCBI data base and aligned. The putative BCL6 binding sites are indicated by the yellow boxes; +1 indicates the transcription start site.

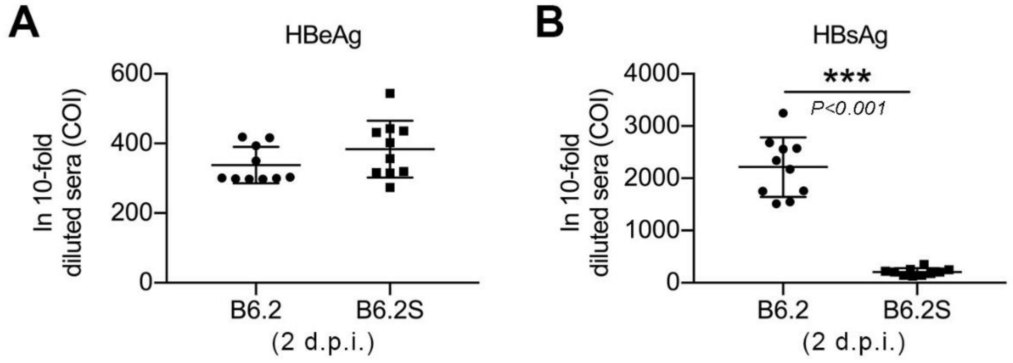

Supplementary Figure S5. B6.2S secretes much lower levels of HBsAg than B6.2 into serum. (**A**) The serum levels of HBeAg measured on day 2 post-DNA injection showed that the HDI efficiencies between the B6.2 and the B6.2S groups were comparable. (**B**) The serum levels of HBsAg in the B6.2S group were much lower than those in the B6.2 group. ***P<0.001, the Student's t-test.

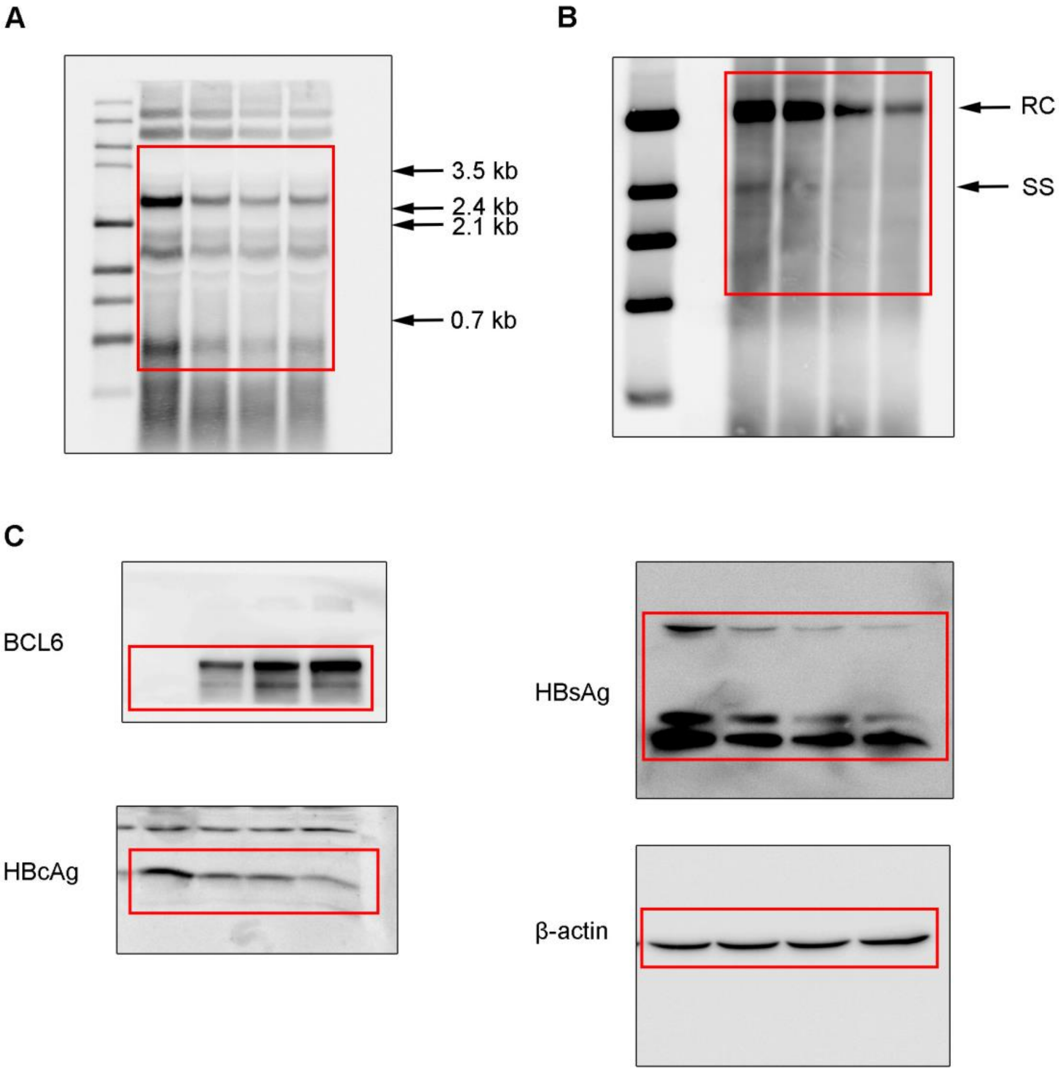

Supplementary Figure S6. Original blot images

(A) For Figure 5B. (**B**) For Figure 5D. (**C**) For Figure 5E.

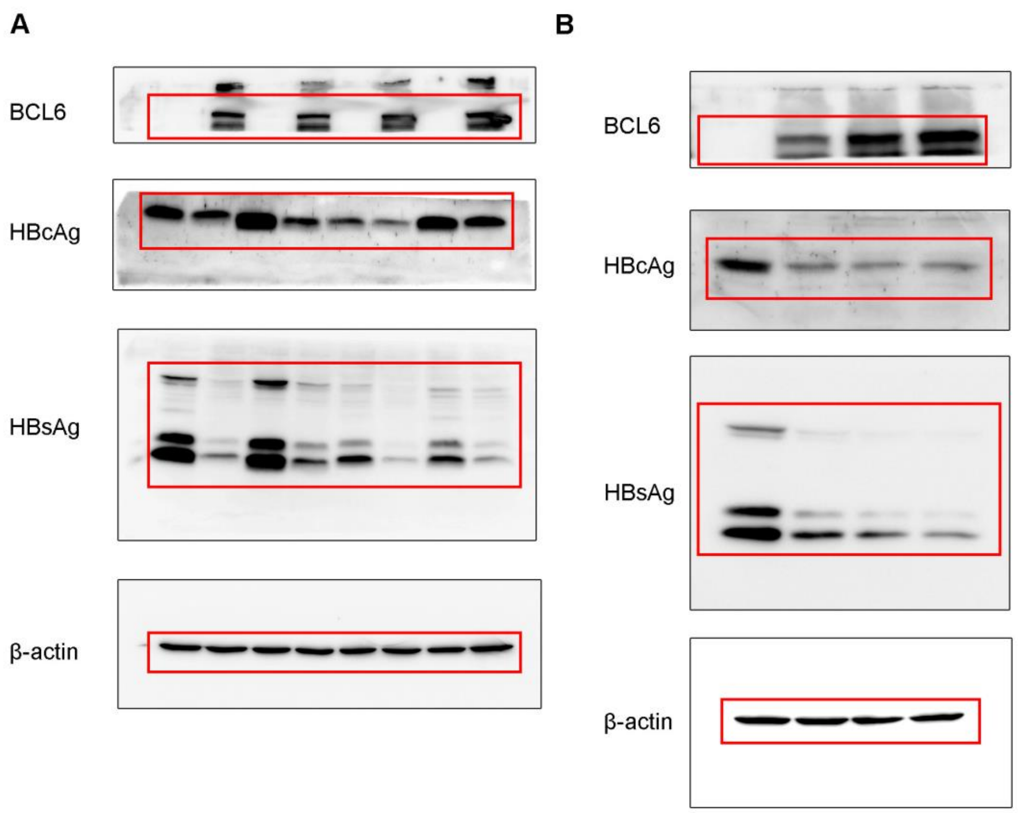

Supplementary Figure S7. Original blot images

(A) For Figure 6B. (**B**) For Figure 6D.
